# Heart ventricles of the dromedary camel (*Camelus dromedarius*): new insights from sectional anatomy, 3D computed tomography, and morphometry

**DOI:** 10.1186/s40850-023-00173-w

**Published:** 2023-08-18

**Authors:** Mohamed A.M. Alsafy, Samir A.A. El-Gendy, Basma M. Kamal, Catrin S. Rutland, Hanan H. Abd-Elhafeez, Soha Soliman, Ahmed N. ELKhamary, Ahmed G. Nomir

**Affiliations:** 1 Anatomy and Embryology Department, Faculty of Veterinary Medicine, Alexandria University, Alexandria, Egypt; 2 Anatomy and Embryology Department, Faculty of Veterinary Medicine, University of Sadat City, Sadat City, Egypt; 3 School of Veterinary Medicine and Science, Faculty of Medicine, University of Nottingham, Nottingham, UK; 4 Department of Cell and Tissues, Faculty of Veterinary Medicine, Assiut University, Assiut, Egypt; 5 Department of Histology, Faculty of Veterinary Medicine, South Valley University, Qena, Egypt; 6 Department of Surgery, Faculty of Veterinary Medicine, Damanhour University, Damanhour, Egypt; 7 Anatomy and Embryology Department, Faculty of Veterinary Medicine, Damanhour University, Damanhour, Egypt

**Keywords:** Camel, Heart ventricles, Anatomy, Computed tomography

## Abstract

Dromedary camel heart morphology is a crucial research topic with clinical applications. The study aims to understand the dromedary camel anatomy, morphology, and architecture of the ventricular mass. Results: Sagittal and transverse gross sections were compared to sagittal, transverse, and 3D render volume reconstruction computed tomography (CT) scans. The subepicardial fat, which covered the heart base, the coronary groove (*sulcus coronarius*), the left longitudinal interventricular groove (sulcus *interventricularis paraconalis*), and the right longitudinal interventricular groove (*sulcus interventricularis subsinuosus*), had a relatively low density with a homogeneous appearance in the 3D render volume CT. The pericardium in the color cardiac window was identified better than the black and white window (ghost). Transverse and sagittal CT scans demonstrated the internal structures of the heart, including the right atrioventricular orifice (*ostium atrioventriculare dextrum*), right atrioventricular orifice (*ostium atrioventriculare sinistrum*), and aortic orifice (*ostium aortae*), *chordae tendineae*, the cusps of the valves (*cuspis valvae*), and the papillary muscles (*musculi papillares*). The papillary muscle (*musculi papillares*) was presented with a more moderate density than the rest of the heart, and the cusps of the valves (*cuspis valvae*) had a lower density. The ventricular wall (*margo ventricularis*) exhibited different densities: the outer part was hyperdense, while the inner part was hypodense. The thicknesses of the ventricular mural wall and the interventricular septum (*septum atrioventriculare*) were highest at the midpoint of the ventricular mass, and the lowest value was present toward the apical part. The coronary groove (*sulcus coronarius*) circumference measured 51.14 ± 0.72 cm, and the fat in the coronary groove (*sulcus coronarius*) (56 ± 6.55 cm^2^) represented 28.7% of the total cross-sectional area. Conclusion: The current study provided more information about ventricular mass measurements by gross and CT analysis on the heart, which provides a valuable guide for future cardiac CT investigations in camels in vivo.

## Introduction

Dromedary camels possess a unique adaptability that allows them to survive harsh conditions with minimal sustenance [1–5]. *Camelids*, in general, have adapted well to extreme environments ranging from desert to ice and poor grazing areas [6].

The heart morphology of dromedary camels is an intriguing topic of research. Camels are not only used as draught and food production animals but also as zoo, companion, tourism, and valued athletic and show animals. Diagnosing heart conditions in camels has traditionally been quite complex due to the frequent absence of clinical signs of heart failure [7]. It is still debatable whether the precise architecture and orientation of the ventricular myocardium are important to cardiac functions in health or disease [3, 8]. It is well understood that there is a link between the size of the heart and the propensity to develop and sustain fibrillation [9]. Atrial and ventricular fibrillation are more easily induced and maintained in larger hearts than in small ones [9]. The thickness of the heart wall varies as well; for example, the outer wall of a ventricle is significantly thicker than that of an atrium, and the outer wall of the left ventricle is thicker than that of the right ventricle [8, 10]. The myocardial fiber structure in the left ventricle plays a critical role in determining mechanical properties, such as ventricular torsion, strain, and stress [8, 10].

Computed tomographic scanners can display anatomic structures in the heart and great vessels with remarkable fidelity and provide cross-sectional displays. Still, they can also display any selected reconstructed image in oblique, cross, or sagittal sections using computer manipulation [11, 12]. Computed tomography can identify the wall thickness and total myocardial mass [13, 14]. Cardiac CT has increasingly become an alternative imaging modality for assessing left ventricular mass [15].

The current study provides a more detailed investigation into the anatomy, morphology, and architecture of the ventricular mass in the dromedary camel using sagittal and cross-sectional heart scans, and 3D render volume reconstructions from computed tomography images, compared to gross anatomical observations.

## Materials and methods

### Animals

Six healthy adult camels aged 6 to 9 years old with no history of clinical cardiac abnormalities were slaughtered by a professional veterinarian in a local slaughterhouse. The animals were slaughtered for meat consumption, not for research purposes. The hearts were extracted at the slaughterhouse, placed on ice, and immediately transferred to the laboratory. CT was imaged within two hours of death on two camel hearts to avoid postmortem changes. The hearts were imaged in the remaining four samples and then placed in freezers to create gross cross-sectional and sagittal sections. The average weight of the hearts was 3.1 ± 0.189 kg.

### Computed tomography (3D render volume CT; 128-slice multi-detector CT scanning protocol)

Two hearts were wrapped in a secure foam to avoid movement interfering with image quality. Each heart was positioned on its right surface for each scan. The CT scans were carried out on an AMDCT scanner with 128 detectors (Aquilion; Toshiba Medical Systems, Tokyo, Japan), with a rotation time of 300 ms and a slice collimation of 128 ± 0.6 mm^2^, using a continuous helical scan mindose technique. Following the acquisition of a preliminary image to determine the 3D-CTA scan range, serial cross-section scans from the apex to the base of each heart were performed at 1.3 mm intervals using a slide thickness of 1 mm. The following soft window settings: mAs 240, Kv 130, W.342 L.52, and the corresponding gross cross-sections of the heart were identified for comparison [16–18].

To obtain sagittal sections using the soft tissue window, the scans were conducted at the level of the interventricular septum, and many scans were made with 1.8 mm intervals at a thickness of 8 mm, with settings set at mAs 240, Kv 130, W.342 L.52, and the corresponding gross sagittal sections of the heart were identified for comparison. CT image reconstruction was undertaken using the optimal reconstruction parameters for hearts. Initially, a set of cross-sectional tomography slices were constructed individually; then, the images were stacked together sequentially to obtain 3D image models of each heart. The reconstruction algorithm was used within Octopus software, which was then converted into a DICOM format, yielding two reconstruction images: ghost render (black and white) and color render (red) [19, 20].

### Sectional anatomy

The four frozen camel hearts were used for either cross or sagittal sectioning. They were serially cut from the base to the apex at 2 cm intervals with a band saw (cross and sagittal sections, nine sections per heart labeled (L1-9); for descriptions of the anatomical structures observed on the sagittal CT scans, please see reference [21, 22]. The slices were sequentially numbered, gently cleaned, and photographed immediately, with the dorsal surface of each slice section facing the camera [23].

### Morphometry of the ventricular mass

The ventricular widths, circumferences, and thicknesses from six camel hearts were all measured in cm (mean, standard deviation). Each gross section level and corresponding CT scan from each specimen had two ventricle measurements taken. The fat and ventricular mass areas were measured in cm^2^. The CT and gross images were analyzed with the ImageJ 1.53 k application (National Institutes of Health, USA) to perform the many measurements undertaken on the heart ventricles using the previously described methods [24–26].

## Results

### General external features of the camel heart ventricles

**Fig. 1:**
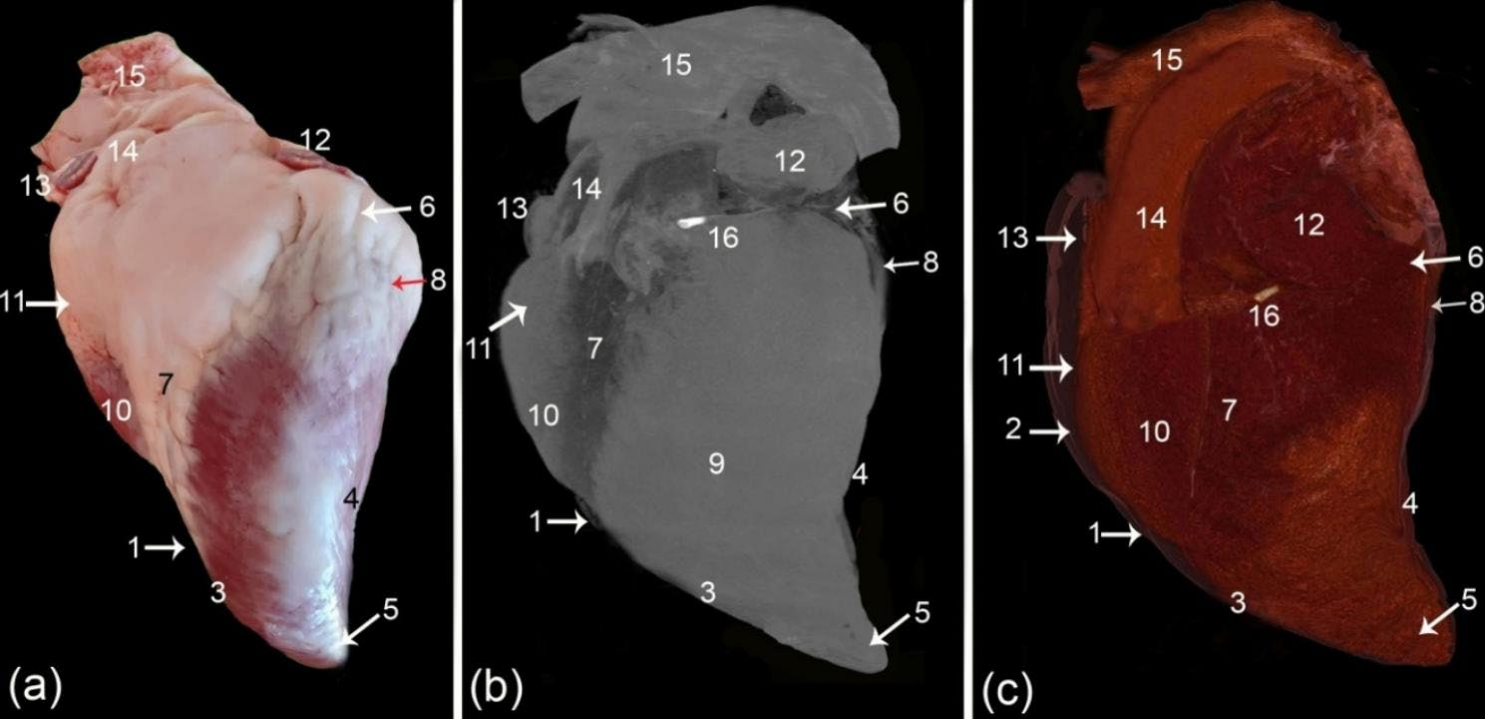
External gross morphology (**a**), and the 3D render volume using the black and white window (**b**) and the colored CT window (**c**) of the camel heart, left view. (1) pericardium, (2) pericardial cavity (*cavum pericardii*), (3) cranial border (*margo ventricularis dexter*), (4) caudal border (*margo ventricularis sinister*), (5) heart apex (*apex cordis*), (6) coronary groove (*sulcus coronarius*), (7) left longitudinal interventricular groove (sulcus *interventricularis paraconalis*), (8) intermediate interventricular groove (*sulcus interventricularis intermedius*), (9) left ventricle (*ventriculus sinister*), (10) right ventricle (*ventriculus dexter*), 11. conus arteriosus, 12. left auricle (*auricula sinister*), 13. right auricle (*auricula dextra*), 14. pulmonary artery (*truncus pulmonalis*), 15. aortic arch (*arcus aorta*), and 16. Ossa cordis dextrum

**Fig. 2:**
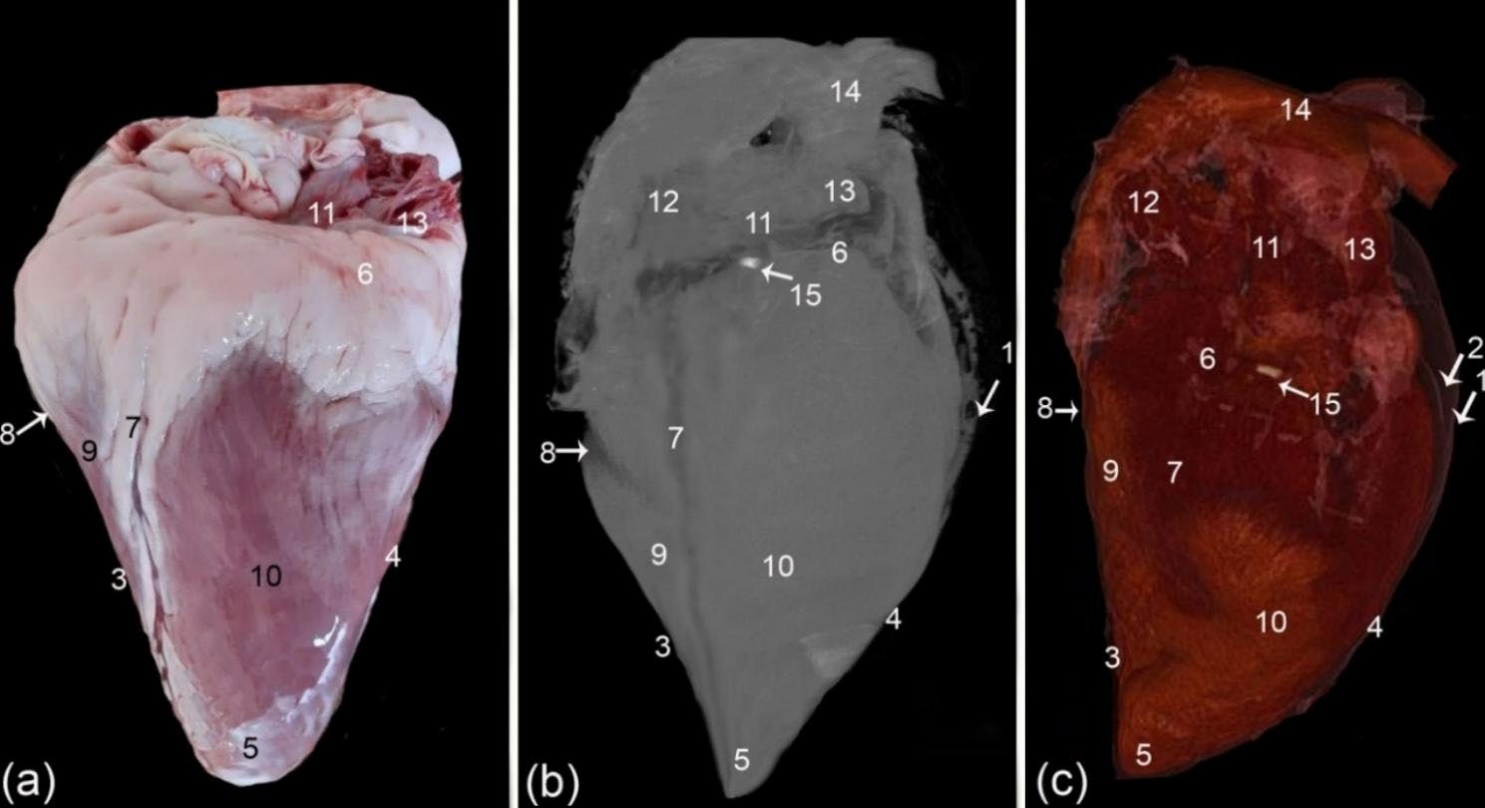
External gross morphology (**a**), 3D render volume using the CT black and white window (**b**) and the colored CT window (**c**) of the camel heart, right view. (1) pericardium, (2) pericardial cavity (*cavum pericardii*), (3) cranial border (*margo ventricularis dexter*), (4) caudal border (*margo ventricularis sinister*), (5) heart apex (*apex cordis*), (6) coronary groove (*sulcus coronarius*), (7) right longitudinal interventricular groove (*sulcus interventricularis subsinuosus*), (8) intermediate interventricular groove (*sulcus interventricularis intermedius*), (9) left ventricle (*ventriculus sinister*), (10) right ventricle (*ventriculus dexter*), 11. right atrium (*atrium dextrum*), 12. cranial vena cava (*venae cavae cranialis*), 13. caudal vena cava (venae cavae caudalis), 14. aortic arch (arcus aorta), and 15. Ossa cordis dextrum

**Fig. 3:**
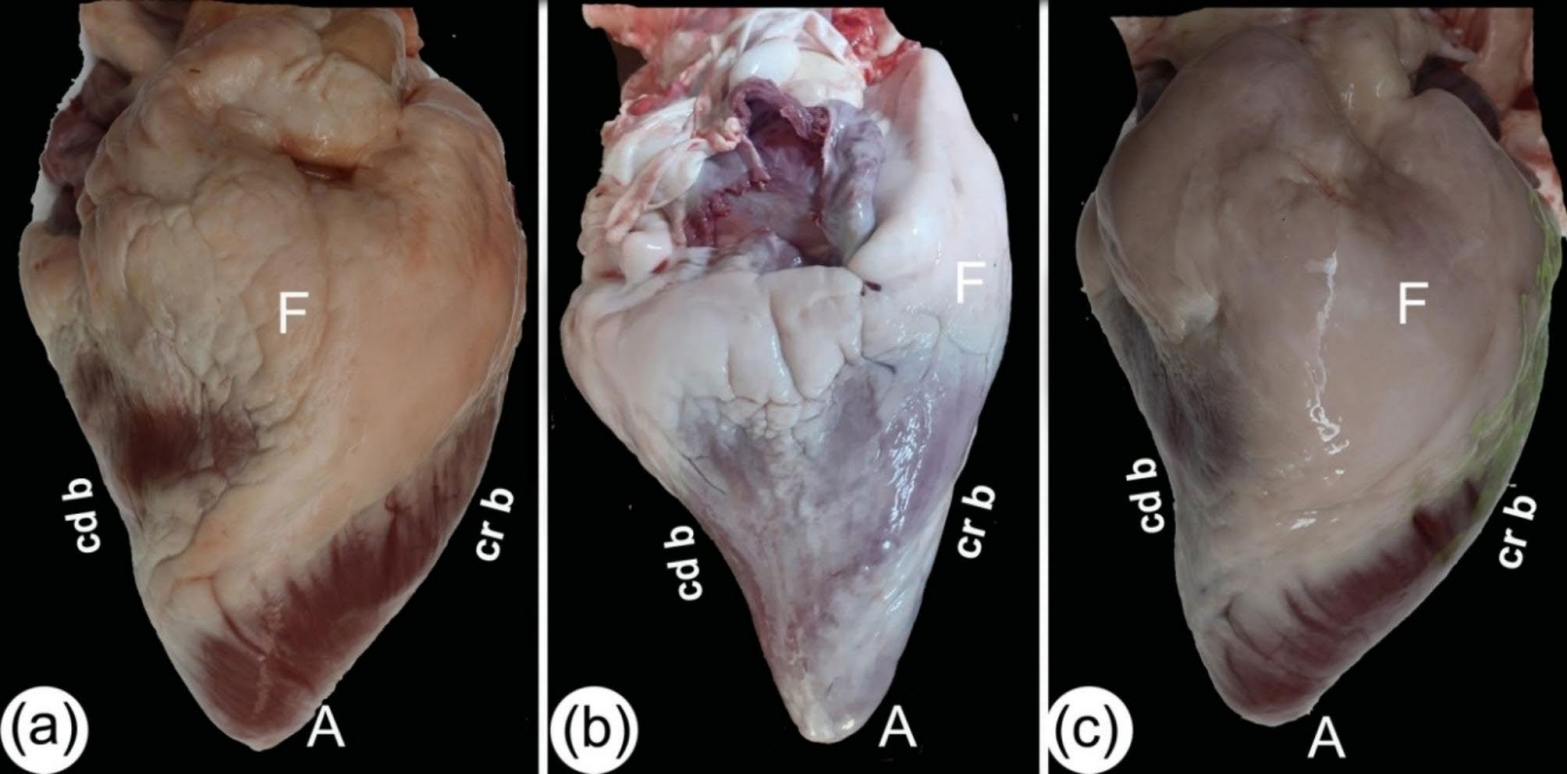
Caudo-right view of the heart in a 6-year-old male (**a**) and a 9-year-old female (**b-c**). The caudal border (*margo ventricularis sinister*) was slightly concave, the cranial border (*margo ventricularis dexter*) was somewhat concave, and the apex (*apex cordis*) was pointed. Fat (F), caudal border (*margo ventricularis sinister*) (cd b), cranial (*margo ventricularis dexter*) (cr b), and heart apex (*apex cordis*) (A)

From the camel heart 3D render volume reconstruction figures, the general morphology of the heart was shown to have an elongated cone shape (Figs. 1 and 2, and 3), with a curved apex that flattened laterally. The external surface of the camel heart contained a large amount of subepicardial fat, which covered the base of the heart, as well as the coronary groove (*sulcus coronarius*), left longitudinal interventricular groove (sulcus *interventricularis paraconalis*), and right longitudinal interventricular groove (*sulcus interventricularis subsinuosus*), with a homogeneous low density exhibited in most cases. In the gross sections, the specimens had a large amount of fat covering the entire heart to varying degrees (Fig. 3/b).

The pericardium was visible in the cardiac color render images, but visualization was optimized in the black and white render image (ghost; Figs. 1 and 2). The ghost black-and-white rendered image also effectively depicted the coronary groove (*sulcus coronarius*), intermediate interventricular groove (*sulcus interventricularis intermedius*), and right longitudinal groove (*sulcus interventricularis subsinuosus*).

**Fig. 4:**
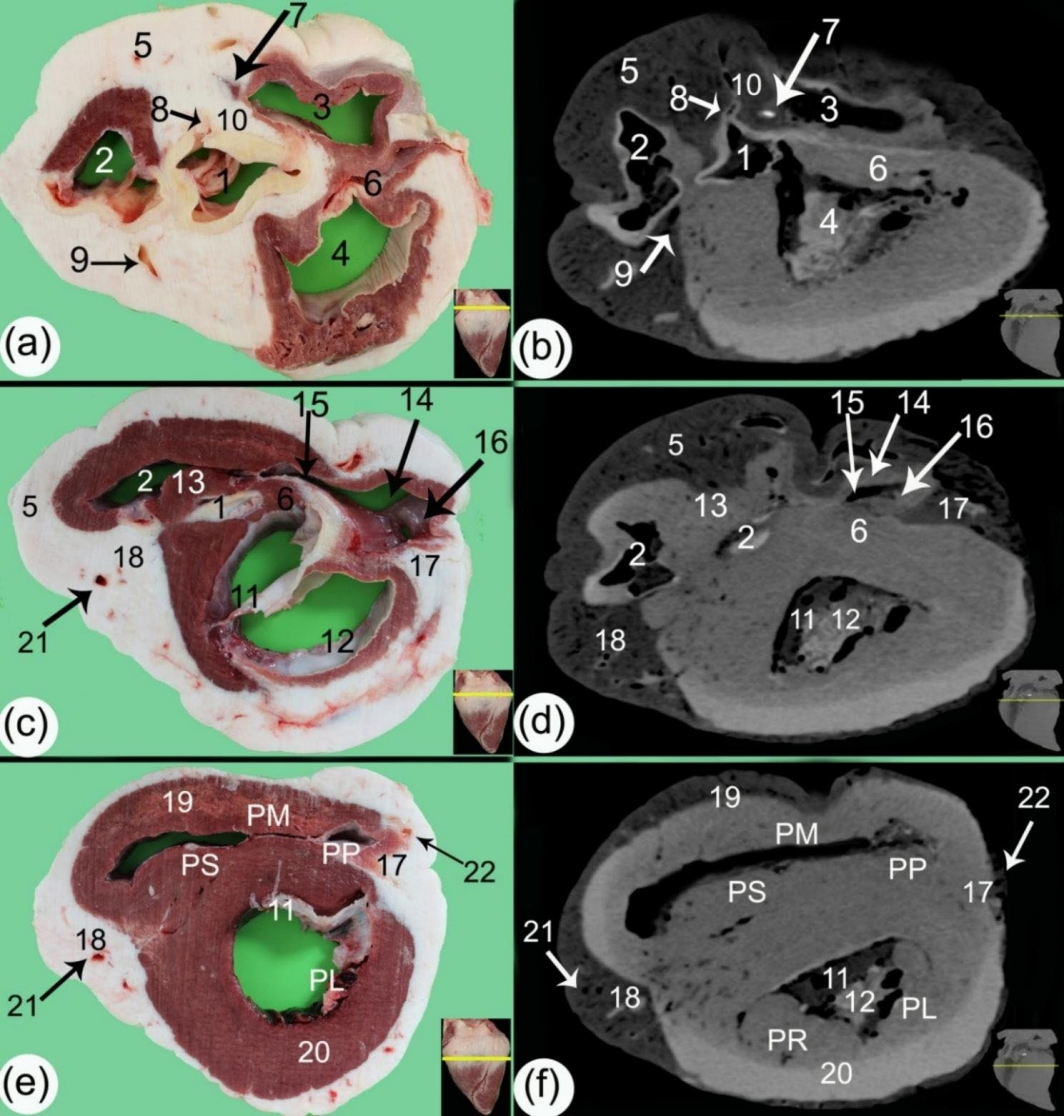
Transverse gross sections (**a, c, e**) and soft tissue window CT scans (**b, d, f**) at the proximal part of the ventricular mass. (1) aorta, (2) conus arteriosus, (3) right atrioventricular orifice (*ostium atrioventriculare dextrum*), (4) left atrioventricular orifice (*ostium atrioventriculare sinsitrum*), (5) subcardinal fat on the coronary groove (*sulcus coronarius*), (6) interventricular septum (*septum atrioventriculare*), (7) ossa cordis dextrum, (8) right coronary artery (*A. coronaria dextra*), (9) left coronary artery (*A. coronaria sinistra*), (10) fibrous area, 11. septal cusp of the bicuspid valve (*cuspis septalis* of *valva atrioventricularis sinistra*), 12. parietal cusp of the bicuspid valve (*cuspis parietalis* of *valva atrioventricularis sinistra*), 13. supraventricular crest (*crista supraventricularis*), 14. parietal cusp of the tricuspid valve (*cuspis parietalis* of *valva atrioventricularis dextra*), 15. septal cusp of the tricuspid valve (*cuspis septalis* of *valva atrioventricularis dextra*), 16. angular cusp of the tricuspid valve (*cuspis angularis*), 17. right subsinosal groove (*sulcus interventricularis subsinuosus*), 18. left paraconal groove (*sulcus interventricularis paraconalis*), 19. right ventricular wall, 20. left ventricular wall, 21. Left paraconal artery (*ramus interventricularis paraconalis*), 22. Right subsinosal artery (*ramus interventricularis subsinuosus*), Pm. *musculus papillaris magnus*, Pp. *musculus papillaris parvi*, Ps. *musculus papillaris subarteriosus*), Pl. left papillary muscle, and Pr. right papillary muscle

The camel heart apex was somewhat elongated, slightly pointed, and round. It was compressed laterally and covered with a slight to moderate amount of adipose tissue. The heart cranial border (*margo ventricularis dexter*) was slightly convex; its upper half was formed by the right ventricle (*ventriculus dexter*) (Fig. 1). The lower half was significantly convex, and it was formed by the left ventricle (*ventriculus sinister*), measuring on average 27.9 ± 0.35 cm, and crossed by a left longitudinal interventricular groove (*sulcus interventricularis paraconalis*) (Fig. 1). The left ventricle (*ventriculus sinister*) formed the left ventricular border (caudal border) (*margo ventricularis sinister*), which varied in shape; it was nearly straight in the upper region, whereas the lower part was concave with a blunt end, the upper part was slightly concave, and the lower part was slight convex (Fig. 4) and was shorter than the right one at 20.5 ± 0.37 cm. The ventricular mass was measured at 21.1 ± 0.4 cm from the apex to the coronary groove (*sulcus coronarius*).

The left longitudinal interventricular groove (*sulcus interventricularis paraconalis*) was observed in the images at the cranial aspect of the left surface (Fig. 1). It originated from the coronary groove (*sulcus coronarius*) at the beginning of the pulmonary artery (*truncus pulmonalis*), reached the right ventricular border (*margo ventricularis dexter*), then continued to the apical part, measuring around 26.8 ± 0.28 cm in total. It divided the heart into proximal quadrilateral and long, slender oblique parts. The right longitudinal interventricular groove (*sulcus interventricularis subsinuosus*) was in the middle part of the arterial surface, extending from the coronary groove (*sulcus coronarius*) proximally below the termination of the caudal vena cava (*venae cavae caudalis*) in the right atrium (*atrium dextrum*) (Fig. 2). It descended vertically towards the apex of the heart, measuring between 17 and 18 cm (17.58 ± 0.17), which was shorter than the left longitudinal interventricular groove (*sulcus interventricularis paraconalis*). The intermediate interventricular groove (*sulcus interventricularis intermedius*) was a small and short groove extending from the coronary groove (*sulcus coronarius*) down to the caudal border on the left side (Fig. 1).

### Sectional anatomy and CT scans

**Fig. 5:**
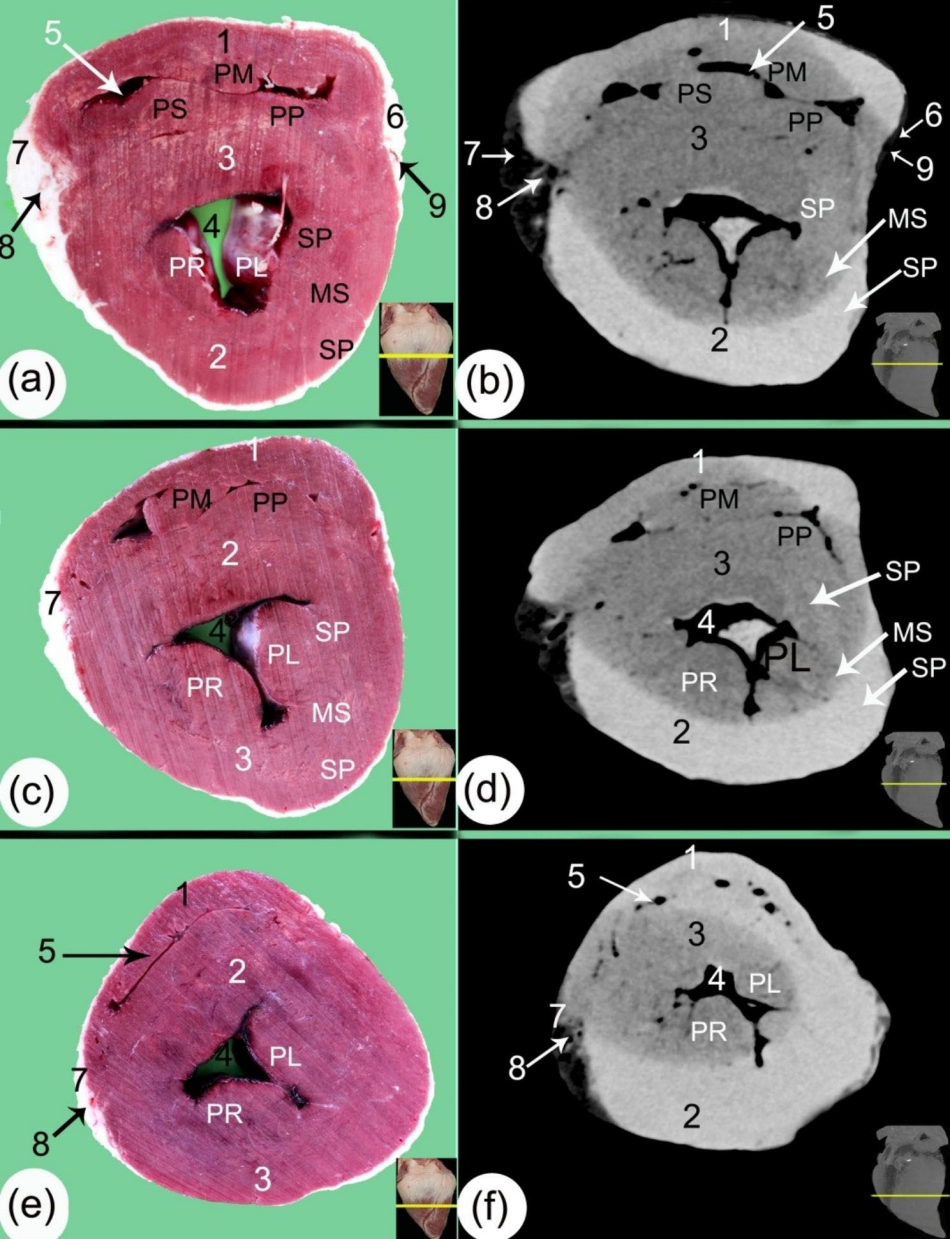
Transverse gross sections (**a, c, e**) and soft tissue window CT scans (**b, d, f**) in the middle part of the ventricular mass. (1) right ventricular wall (*margo ventricularis dexter)*, (2) left ventricular wall (*margo ventricularis sinister*), (3) interventricular septum (*septum atrioventriculare*), (4) left ventricular cavity, (5) right ventricular cavity, (6) right subsinosal groove (*sulcus interventricularis subsinuosus*), (7) left longitudinal interventricular groove (*sulcus interventricularis paraconalis*) containing a moderate amount of fat, (8) left paraconal artery (*ramus interventricularis paraconalis*), (9) right subsinosal artery (*ramus interventricularis subsinuosus*), Pm. *musculus papillaris magnus*, Pp. *musculus papillaris parvi*, Ps. *musculus papillaris subarteriosus*, Pl. left papillary muscle, Pr. right papillary, SP. subepicardial layer, MC. mesocardial layer, and SN. subendocardial layer

**Fig. 6:**
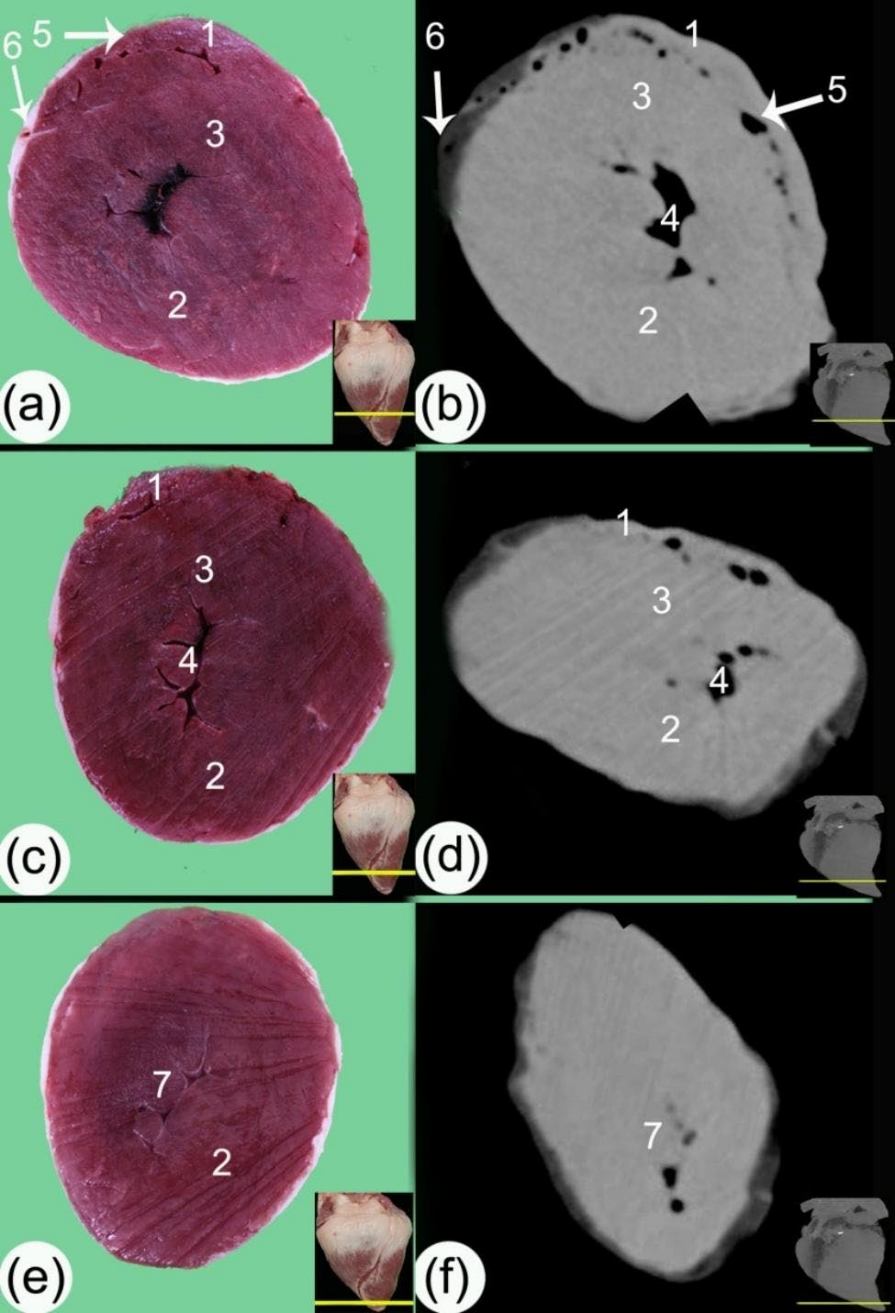
Transverse gross sections (**a, c, e**) and soft tissue window CT scans (**b, d, f**) at the ventral part of the ventricular mass. (1) right ventricular wall (*margo ventricularis dexter)*, (2) left ventricular wall (*margo ventricularis sinister*), (3) interventricular septum (*septum atrioventriculare*), (4) left ventricular cavity, (5) right ventricular cavity, (6) left paraconal groove (*sulcus interventricularis paraconalis*), and (7) trabeculae carneae

**Fig. 7:**
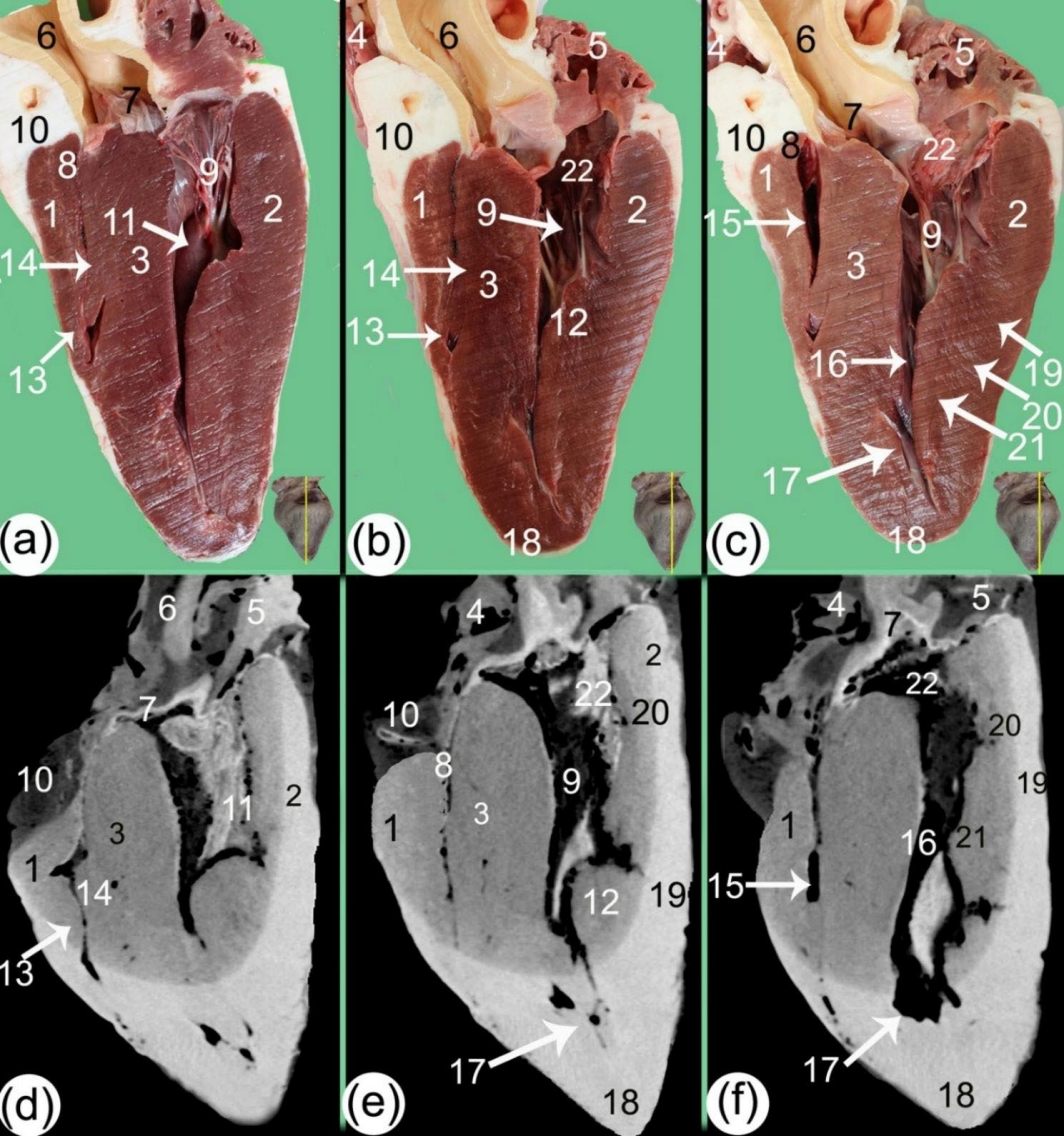
Sagittal gross sections (**a, b, c**) and soft tissue window CT scans (**d, e, f**) of the camel heart at the level of the aorta exhibiting the ventricular structures. 1. mural wall of the right ventricle (*ventriculus dexter*), (2) mural wall of the left ventricle (*ventriculus sinister*), (3) interventricular septum (*septum atrioventriculare*), (4) right auricle (*auricula dextra*), (5) left auricle (*auricula sinister*), (6) aorta, (7) aortic orifice (*ostium aortae*), (8) right atrioventricular orifice (*ostium atrioventricular dextrum*), (9) chordae tendineae, (10) subcardinal fat, 11. right papillary muscle, 12. left papillary muscle, 13. *musculus papillaris magnus*, 14. *musculus papillares parvi*, 15. right ventricular cavity, 16. left ventricular cavity, 17. trabeculae carneae, 18. heart apex (*apex cordis*), 19. subepicardial layer, 20. mesocardial layer, 21. subendocardial layer, and 22. cusps

The transverse and sagittal gross sections of the hearts were compared to the CT scans. The heart base (*basis cordis*) had a somewhat quadrilateral outline (Figs. 1, 2, 3, 4 and 5). The fat had a lower density than the rest of the heart tissue and was present mainly in the atrial region (Figs. 3 and 4). A large amount of fat was present at the circumference of the coronary groove (*sulcus coronarius*) and had a depth of about 2.5–4 cm (3.2 ± 0.24) on the left longitudinal interventricular groove (*sulcus interventricularis paraconalis*), reduced to 1.5–2.8 cm (2.04 ± 0.22) on the right longitudinal interventricular groove (*sulcus interventricularis subsinuosus*) (Figs. 2, 3 and 4). The right ventricle (*ventriculus dextrum*) had a crescent-type shape when viewed under transverse CT; its mural side was concave, and its septal side was convex and bulged into the cavity (cross-section views; Figs. 4 and 5, and 6). It was possible to visualize the papillary muscle (*musculi papillares*), supraventricular crest (*crista supraventricularis*), conus arteriosus, and pulmonary trunk orifice (*ostium trunci pulmonalis*) (Figs. 4 and 5, and 7).

In the sagittal planes of the CT scans, the right atrioventricular orifice (*ostium atrioventriculare dextrum*), chordae tendineae, and cusps were also visible (Fig. 7). The left ventricle (*ventriculus sinister*) appeared triangular at its proximal part and had an irregular, circular shape in the middle part in the transverse CT scans (Figs. 4 and 5). In both the sagittal and cross-sectional views, the left atrioventricular (*ostium atrioventriculare sinistrum*) orifice and aortic (*ostium aortae*) orifice were demonstrated. The papillary muscle (*musculi papillares*) appeared to have a moderate density in comparison to the rest of the heart, and the cusps had a lower density appearance than the papillary muscles (*musculi papillares*) (Fig. 4).

The mural wall had differing densities in the outer, middle, and inner parts; the outer layer was hyperdense, and the inner layer was hypodense; these were the layers of myocardial muscle fibers, the subepicardial layer, the mesocardial layer, and the subendocardial layer (Figs. 5 and 7). The trabeculae carneae were present mainly in the ventral part of the left ventricles (Fig. 5).

### Morphometry of the ventricular mass

The heart was measured at nine different levels: three in the upper ventricular part, three in the middle, and three in the lower ventricular part, at about 2 cm intervals, with the resulting measurements shown in Table 1, the measurements of the ventricular wall using the CT images and ImageJ. The maximum width between the two longitudinal grooves was 17.52 ± 0.16 cm, nearly at the level of the coronary groove (*sulcus coronarius*). The circumference of the heart at the apex ranged from 51.14 ± 0.72 cm in the coronary groove (*sulcus coronarius*) to 6.36 ± 0.17 cm at the distal apical cross-section. The thickness of the mural wall of the ventricles and the interventricular septum (*septum atrioventriculare*) were highest at the middle ventricular mass, and the lowest value was present toward the apical part. The left ventricle (*ventriculus sinister*) thickness ranged from 0.95 ± 0.04 cm at the apical part to 4.36 ± 0.44 cm at the middle ventricular part; the right ventricle (*ventriculus dexter*) thickness ranged from 0.38 ± 0.02 cm at the ventral part to 1.92 ± 0.21 cm at the middle ventricular part; and the interventricular septum (*septum atrioventriculare*) thickness ranged from 1.88 ± 0.07 cm to 3.58 ± 0.30 cm. The ventricular circumference ranged from 6.36 ± 0.17 cm to 51.14 ± 0.72 cm. The fat area in the coronary groove (*sulcus coronarius*) occupied 56.00 ± 6.55 cm^2^, which was 28.7% of the total area of the cross-section at these levels, and the area above it between the valves of the heart measured up to 70.80 ± 3.55 cm^2^. It resembled about 45% of the total area of the cross-section at these levels. At the same time, on the apex, it was reduced to 1.38 cm^2^, and the whole area of the heart near the coronary groove (*sulcus coronarius*) was 194.24 ± 4.40 cm^2^ (Table 1).

**Table 1:** Morphometry of the ventricular mass (mean ± standard deviation)

Level	Width between grooves (cm)	Width between borders (cm)	Ventricular mass circumference (cm)	Total area (cm^2^)	Fat area (cm^2^)	R. ventricle thickness (cm)	L. ventricle thickness (cm)	Interventricular septum (cm)
**L1**	2.08 ± 0.04	2.10 ± 0.05	6.36 ± 0.17	4.90 ± 0.40	0.19 ± 0.06	---------	0.95 ± 0.04	---------
**L2**	7.20 ± 0.52	7.68 ± 0.44	25.00 ± 0.29	43.54 ± 2.23	1.38 ± 0.20	0.38 ± 0.02	2.86 ± 0.22	1.88 ± 0.07
**L3**	9.55 ± 0.05	9.70 ± 0.15	31.28 ± 0.21	73.64 ± 0.99	3.46 ± 0.33	0.55 ± 0.09	3.92 ± 0.41	2.00 ± 0.16
**L4**	10.80 ± 0.27	11.4 ± 0.24	36.64 ± 1.25	97.46 ± 4.75	5.53 ± 0.16	0.96 ± 0.04	4.02 ± 0.53	3.01 ± 0.05
**L5**	12.80 ± 0.05	13.20 ± 0.06	42.94 ± 0.66	134.90 ± 1.63	8.88 ± 0.15	1.88 ± 0.30	4.36 ± 0.44	3.22 ± 0.05
**L6**	14.87 ± 0.4	13.20 ± 0.29	44.53 ± 1.33	147.80 ± 7.48	20.24 ± 1.10	1.92 ± 0.21	3.22 ± 0.32	3.58 ± 0.30
**L7**	16.50 ± 0.07	14.60 ± 0.15	49.60 ± 0.17	176.74 ± 0.58	35.68 ± 3.82	1.812 ± 0.3	2.85 ± 0.29	3.10 ± 0.21
**L8**	17.52 ± 0.16	15.10 ± 0.08	51.14 ± 0.72	194.24 ± 4.40	56.00 ± 6.55	1.59 ± 0.19	2.82 ± 0.28	3.00 ± 0.07
**L9**	16.64 ± 0.07	14.77 ± 0.04	50.20 ± 0.58	190.00 ± 3.03	70.8 ± 3.55	1.46 ± 0.02	3.44 ± 0.17	2.62 ± 0.19

## Discussion

According to our sources, this is the first paper to use CT to examine ventricular mass, determine the main structures and their measurements at many levels of the heart, and create a 3D rendering of adult camel hearts. The camel’s neck is long curved, which allows it to reach high plants and see further distances across its natural habitat of the desert [27]. Because the long neck and legs require a powerful pump to push blood to the brain, we focused our research on the ventricular mass of the heart. The ability of computed tomography to identify the inner endocardial walls and the epicardial surface distinguishes it. As a result, while wall thickness and total myocardial mass are essential [11, 14], early results revealed a close relationship between computed tomographic measurements and postmortem anatomic measurements. In dogs, the in vivo left ventricular mass correlated well with autopsy measurements. The 3-D modalities of cardiac CT were appropriate techniques for assessing left ventricular functional measures and providing a good description of the cardiac structures [28–30].

The reconstructed CT render volume work in the present study showed the camel heart in 3D as an elongated cone shape with a curved, pointed apex flattened laterally with a large amount of subepicardial fat. The heart appeared conical and pointed, with extensive fat deposition, notably on the external surface and around the coronary groove (sulcus coronarius), similar to previous studies [31–33]. In comparison, the heart of an alpaca, which is in the same order as the camel, is inflated and pointed in a conical shape, with its apex pointing caudally and ventrolaterally [34].

In the present study, the amount of fat and ventricular wall thickness was also measured in camels using these CT scanning methods at different points throughout the heart. MRI nowadays is the gold standard imaging modality for most body tissues, especially fatty liver disease [35]. CT may provide a more accurate assessment of fat content because fat has a higher spatial resolution than MRI and ultrasound [36, 37]. In our study, the fat had a homogeneous low density when visualized using CT and accumulated at the coronary groove (*sulcus coronarius*) and longitudinal grooves (*sulci interventricularis*). More fat was present at the left longitudinal groove (*sulcus interventricularis paraconalis*), where the fact was white and occupied 28.7% of the total area of the cross-section at the coronary groove (*sulcus coronarius*) level. This result was nearly identical to that previously observed via gross anatomical dissection in the camel [33, 38]. The camel has an unusually high amount of fat that fills the coronary (*sulcus coronarius*) and longitudinal grooves (*sulci interventricularis*), and this was especially noted previously between the atria [38]. An accumulation of fat has been pointed out on the left side, and fat comprises about 29.03% of the weight of the heart [39].

3D CT was used in the present study to outline the ventricular external futures. The CT sections provided more detailed information about external and internal structures and measurements. Previous studies using CT in other species, such as the elephant ex vivo, revealed that the left lateral ventricular wall thickness was 5 to 6 cm, the right ventricular wall thickness was 1.5.25 cm, and the atria possessed a wall of 15–30 mm [40]. This indicates that it could be a valuable technique to explore in vivo for clinical use.

The heart circumference ranged from 51.14 ± 0.72 cm at the coronary groove (*sulcus coronarius*) to 6.36 ± 0.17 at the distal apical cross-section. The present study detailed several measurements throughout the heart using this technique. In contrast, other papers have shown figures using different techniques and were less detailed regarding the number of measures taken. The present work did show comparable measurements, though with others conducting the circumference of the camel heart at the level of the coronary groove (*sulcus coronarius*) was 45 cm [33], 33.02 cm at the coronary groove (*sulcus coronarius*), 2.54 cm nearer the apex [38], and 36.45 ± 0.57 cm at the level of the coronary groove (*sulcus coronarius*) [31].

The mean of measurements across all levels analyzed regarding the right ventricle thickness was 1.13 cm, while at the left ventricular wall (*margo ventricularis sinister*), it was 3.15 cm. Previous echography on a camel aged 8.8 years mentioned these measurements were 1.1–3.4 cm at systole and diastole for the left ventricle, 1.2–2.4 cm for the right ventricle, and 1.2–3.9 cm in the interventricular septum (*septum atrioventriculare*) [41]. The force required during contraction determines the thickness of the heart wall at a specific location [8, 42]. The giraffe, for example, has small cavities and a relatively thick wall, which produced arterial pressures at the normal left ventricular wall (*margo ventricularis sinister*) tension, which was necessary to ensure brain perfusion [43]. The camel’s heart beats at an average of 50 beats per minute, with a blood volume of 93 ml/kg and blood pressure ranging from 76 to 115 mmHg; these values exceeded those of other domestic ruminants [44]. The thickness of the right ventricular wall was greater than that of the left ventricular wall (*margo ventricularis sinister*) in 101-day-old camel embryos [45]. The right ventricular wall was measured at 0.82 cm in camel calves, and the left ventricular wall (*margo ventricularis sinister*) was 1.96 cm. In comparison, the right ventricular wall (*margo ventricularis dexter*) was thin and varied in thickness along its length (0.6–1 cm) [33]. The left ventricular wall (*margo ventricularis sinister*) is three times thicker than the right in camels [33] and in dogs [46, 47]. In contrast, the left ventricle (*ventriculus sinister*) is double the thickness of the right in domestic animals [48, 49]. We believe these differences are due to the relatively thicker ventricle wall than observed in other domestic mammals. Arterial pressure in camels may rise by 300 mmHg, necessitating a large heart. Still, the giraffe’s heart is like that of other mammals in terms of body mass, so the increased thickness of the camel’s ventricular wall may play the same role as the giraffe’s heart.

## Conclusion

This study contributed to our comprehension of ventricular mass measurements in the camel heart ex vivo using gross and CT imaging. It is unique in that this technique can detect the inner endocardial walls and the epicardial surface. Three-dimensional cardiac CT modalities were used to assess ventricular functional measurements and provide a detailed depiction of the cardiac structures.

## Data Availability

The datasets used and analyzed during the current study are available from the corresponding author upon reasonable request.
